# Vascularity of the gastric conduit predicts complications after Ivor-Lewis esophagectomy

**DOI:** 10.1007/s00464-025-11780-8

**Published:** 2025-05-08

**Authors:** Julian Lemties, Carolin Scheidt, Jin-On Jung, Naita M. Wirsik, Leandra Lukomski, Dolores Krauss, Anders Grabenkamp, Alexander R. Stier, Su Ir Lyu, Alexander I. Damanakis, Benjamin Babic, Alexander Quaas, Thomas Schmidt, Hans F. Fuchs, Christiane J. Bruns, Wolfgang Schröder, Lars M. Schiffmann

**Affiliations:** 1 Department of General, Visceral, Cancer and Transplantation Surgery, Faculty of Medicine, University Hospital Cologne, University of Cologne, Kerpener Str. 62, 50937 Cologne, Germany; 2 Institute of Pathology, Faculty of Medicine, University Hospital Cologne, University of Cologne, Cologne, Germany; 3 Department of General and Visceral Surgery, Sana Klinikum Offenbach, Offenbach am Main, Germany

**Keywords:** Esophageal cancer, Esophagectomy, Ischemic conditioning, Postoperative complications, Gastric conduit, Neoangiogenesis

## Abstract

**Background:**

Anastomotic leakage (AL) contributes to postoperative morbidity and mortality after Ivor-Lewis esophagectomy. Vascular high-risk patients show a significantly increased risk of AL. We previously showed that laparoscopic ischemic conditioning (ISCON) of the stomach prior esophagectomy in these high-risk patients is a safe procedure that induces neoangiogenesis at the anastomotic site. Our data also suggested that this directly impacts on anastomotic healing.

To further investigate the hypothesis that gastric conduit vascularization directly influences postoperative morbidity, we evaluated gastric conduit vascularity in a cohort of patients undergoing two-stage esophagectomy prior to the ISCON era.

**Material and Methods:**

Seventy-nine patients who underwent two-stage esophagectomy from 2016 to 2021 at our center were retrospectively analyzed from a prospectively maintained database. Microvessel density (MVD) of the gastric conduit at the anastomotic region was evaluated by CD34 staining of the gastric stapler ring. Analysis of microvessel density (MVD) was performed using ImageJ. Patients were stratified into low- and high-MVD groups, and MVD was correlated with clinical outcomes.

**Results:**

Patients with a high MVD showed a significantly lower rate of anastomotic leakage (AL) in comparison to patients with low MVD (6.25% vs. 22.58% *p*=0.043). Furthermore, a high MVD was associated with a lower rate of major complications (Clavien Dindo ≥ IIIb) (12.50% vs. 38.71% *p*=0.012) and a shorter hospital stay (17.9 days vs. 23.1 days, *p*=0.045).

**Conclusion:**

Vascularization of the stomach might function as surgical biomarker of AL in patients undergoing two-stage esophagectomy. Prospective trials have to further substantiate this finding.

**Supplementary Information:**

The online version contains supplementary material available at 10.1007/s00464-025-11780-8.

Esophageal cancer remains one of the most lethal malignancies worldwide [1]. The standard treatment for locally advanced disease involves multimodal therapy followed by transthoracic esophagectomy with gastric reconstruction, commonly performed as an Ivor-Lewis esophagectomy [2–4]. Despite advancements in perioperative management and surgical techniques, including minimally invasive and robot-assisted procedures, esophagectomy remains a complex intervention with notable morbidity and mortality rates [3, 5] with anastomotic leakage being the most relevant one [5].

Recent studies have identified severe atherosclerosis of the thoracic aorta and celiac axis stenosis as key contributors to inadequate gastric tube perfusion as a patient-related trigger of AL independent of the surgical procedure or intraoperative course [6–9]. The concept of gastric ischemic conditioning aims, to separate the phase of gastric devascularization and the phase of reconstruction in two stages to improve vascular perfusion at the anastomotic site by inducing neoangiogenesis that occurs in the interval of ischemic conditioning [10]. This two-stage esophagectomy can be performed by different techniques. The abdominal part of the Ivor-Lewis esophagectomy can either be performed by a complete gastric mobilization including the abdominal part of the lymphadenectomy with the thoracic part following a few days later [11] (so-called two-stage esophagectomy, which was the standard approach in high-risk patients at our center and subject of this work until enrollment of the ISCON Trial) or the procedure of ischemic conditioning can be performed as a stand-alone procedure with the complete abdominothoracic procedure following 14 days later [12]. We have previously demonstrated that both techniques are safe options to surgically treat patients with severe (vascular) co-morbidities and esophageal cancer. We also showed that there might be a correlation between vascularity of the stomach and anastomotic leakage in these high-risk patients [13–15].

This works aims to further explore whether vascular density of the stomach at the point of the esophagogastric anastomosis is associated with the risk of anastomotic leakage in patients undergoing a two-step esophagectomy.

## Material and methods

### Study design

This retrospective cohort study focuses on patients who underwent Ivor-Lewis esophagectomy for adenocarcinoma or squamous cell carcinoma of the esophagus at the Department of General, Visceral, and Cancer Surgery, University of Cologne, a tertiary care institution, during the period between 2016 and 2021. The indication for surgery and neoadjuvant therapy was determined by an interdisciplinary tumor board prior to the operation. A total of 1012 patients were initially evaluated, with 924 patients who underwent one-stage esophagectomy excluded from the study.

The cohort of interest comprised 88 patients who underwent two-stage esophagectomy due to pulmonary or vascular comorbidities. Following histological analysis, nine patients were excluded due to the absence of gastric mucosa on stained samples, resulting in a final study population of 79 patients.

This retrospective study utilizes data extracted from a prospective database. Ethical approval was obtained from the local Institutional Review Board, with patient consent for data analysis waived as individual patient identification was not possible. The investigation aims to provide insights into the outcomes of two-stage esophagectomy in the specified cohort, contributing valuable information to the existing body of knowledge on esophageal cancer treatments.

### Surgery

The indication for a two-stage esophagectomy was determined preoperatively. Patients were selected for the two-stage procedure based on either vascular or pulmonary comorbidities. Pulmonary indications included advanced pulmonary diseases, such as chronic obstructive pulmonary disease (COPD), or a combination of co-morbidities that led the surgical team doubt the patient’s fitness for a one-stage thoracoabdominal procedure. Vascular indications included extensive peripheral artery disease, stenosis of the celiac trunk, or stenosis of the superior mesenteric artery.

Two-stage hybrid IL esophagectomy consisted of two separated surgical procedures. The first operation included the complete laparoscopic gastric mobilization with abdominal lymphadenectomy. The fatty tissue along the lesser curvature remained untouched and gastric tube formation was not initiated during the abdominal phase. Lymph node dissection was done after incision of the lesser sac along the common hepatic artery and splenic artery, followed by dissection of the left gastric artery with nodal clearance of the retroperitoneal space up to the lower mediastinum (modified D2-lymphadenectomy). The dissected tissue remained attached to the lesser curvature. The gastroesophageal junction was completely mobilized at the level of the diaphragmatic hiatus. Devascularization also included dissection of the short gastric arteries along the gastric fundus. Finally, the greater curvature was completely mobilized with visualization and preservation of the right gastroepiploic vessels.

After an interval of 1 to 7 days right sided open transthoracic esophagectomy was performed with dissection of the mediastinal lymph nodes (2-field lymphadenectomy). A shorter time to reconstruction was chosen in patients primarily with pulmonary comorbidities. For patients diagnosed with squamous cell carcinoma, lymphadenectomy is extended to compartments on the left side of the trachea (extended 2-field lymphadenectomy). After gastric pull-up, the gastric tube is constructed with a width of approximately 4 cm using a longitudinal stapler (45mm and 60mm longitudinal Endo-GIA (Medtronic®) stapling devices). Esophagogastric anastomoses were sutured as an end-to-side anastomosis above the level of the dissected azygous vein with a circular stapler (25mm or 28mm, EEA, Medtronic®). The anastomosis was located at the anterior wall of the gastric corpus close to the greater curvature. After placing several tension-release sutures, the circular anastomosis was covered by an omental flap. Nasogastric tube was placed in the gastric conduit at anastomotic level to rail and relieve pressure from the esophagogastrostomy.

The gastric stapler ring from the region of the gastric fundus, where the anastomosis is created, was forwarded to the Institute of Pathology for microscopic investigation.

### Postoperative management

All patients were extubated in the operating room and subsequently transferred to the intensive care unit (ICU) for postoperative recovery.

Oral feeding was initiated on postoperative day 6, starting with clear liquids. If no complications arose, the oral diet was advanced and completed by postoperative day 11. During the interval between surgery and the commencement of oral feeding, parenteral nutrition was administered. No routine feeding jejunostomy was implanted.

Pylorotomy or pyloroplasty were not routinely performed. In cases of delayed gastric conduit emptying (DGCE), endoscopic intervention with pneumatic pyloric dilatation was performed. In these cases, a Trilumina gastrojejunal tube was placed, and enteral nutrition was started, independent of the postoperative day.

### Microvessel density

In order to visualize intramucosal vessels within the gastric stapler ring, CD34 staining was conducted on paraffin-embedded sections [16]. A mouse monoclonal anti-human-CD34 antibody (dilution 1:700, Cellmarque/CEQBEnd10) was utilized, and the staining process was carried out using a Leica BOND-MAX stainer (Leica Biosystems, Germany) following the manufacturer's protocol, incorporating a citrate-based antigen retrieval protocol. Subsequently, slides were scanned using a slide scanner for further investigations.

Microvessel density (MVD) quantification was undertaken by two blinded investigators. Five images displaying gastric mucosa from different regions of the gastric stapler ring were randomly selected. Images were captured using Aperio ImageScope (Leica Biosystems Pathology Imaging, Wetzlar, Germany) at 20x magnification. ImageJ software (U.S. National Institutes of Health, Bethesda, Maryland, USA, https://imagej.nih.gov/ij/, 1997–2018) was used for further analysis. Threshold analysis was conducted on each microscopic image using a predetermined threshold value. MVD, expressed as area density (in %), was calculated using ImageJ, as previously described [17]. Mean values from the five samples were determined for comprehensive analysis and interpretation. This methodology provides a standardized approach for the quantitative assessment of intramucosal vessels in the context of gastric stapler ring sections.

### Data collection and statistical analysis

This study sourced data from our meticulously maintained prospective database, encompassing diverse parameters such as demographic information (American Society of Anesthesiologists (ASA), Eastern Cooperative Oncology Group (ECOG), Age, Sex, etc.), comorbidities, and tumor-specific details (neoadjuvant therapy, histology, pTNM stage, and tumor localization).

Postoperative complications were systematically documented following the Esophageal Complication Consensus Group (ECCG) definitions [18], with classification based on the Clavien Dindo Score (CD) [19]. Minor complications were designated for those with a CD score ≤ IIIa, while major complications were categorized for scores ≥ IIIb.

**Fig. 1:**
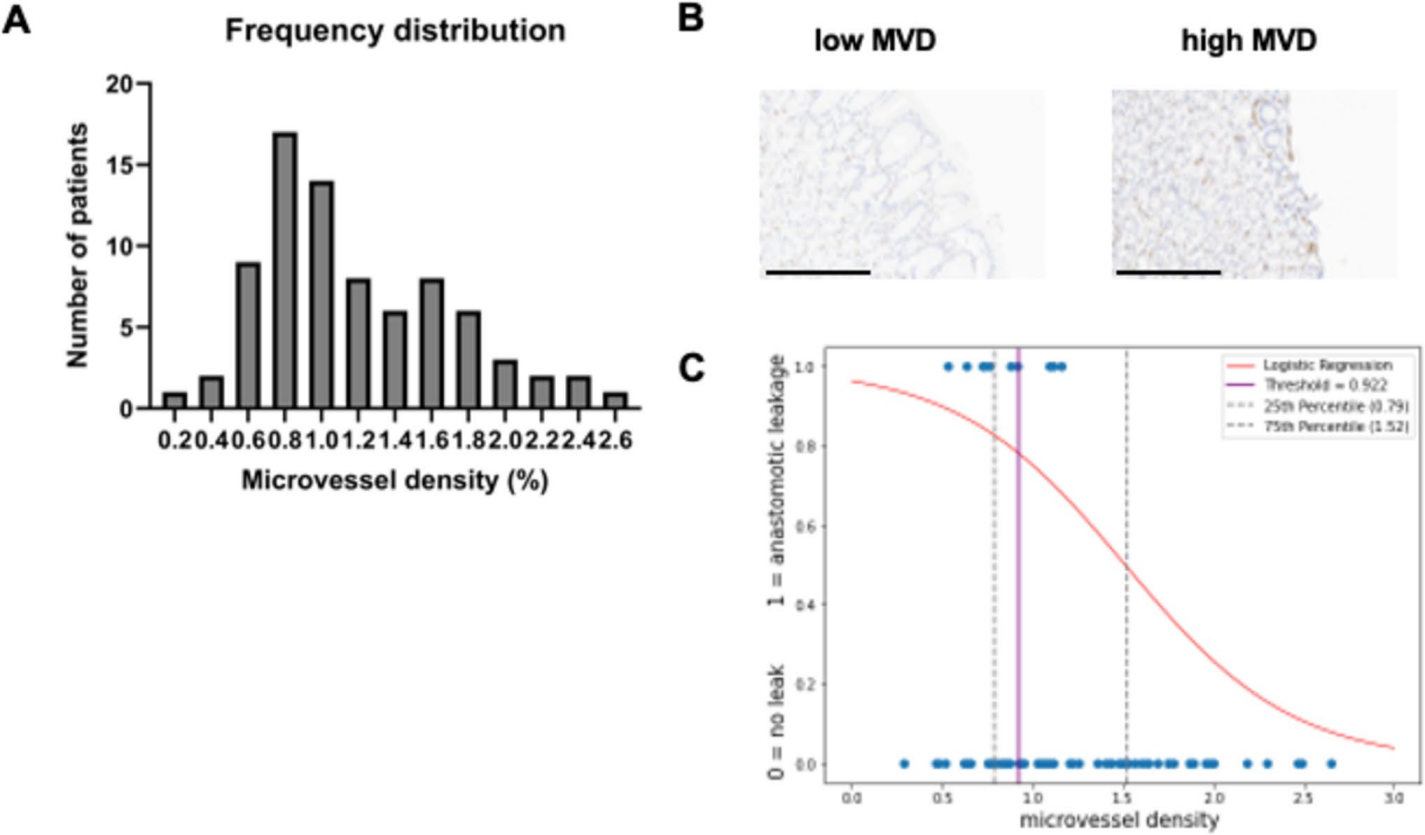
Distribution of MVD and representative images of staining. **A** The histogram illustrates the distribution of Microvessel Density (MVD) values among the groups. **B** Representative images of CD 34 staining of each group. Scale bar equals 300µm. **C** Logistic regression. The optimal cutoff value was calculated via Youden’s index purple line. The logistic regression function is shown as a red sigmoid curve. 25th and 75th percentile is shown as dotted lines (Color figure online)

For statistical analysis, GraphPad Prism 10.1 (GraphPad Software, San Diego, CA, USA) was employed. A *P*-value < 0.05 was considered statistically significant. Standard distribution of microvessel density was assessed using D’Agostino-Pearson test (*p*=0.057, *p*=0.069). A logistic regression model was developed to assess the binary outcome parameter” anastomotic leakage. “The beta coefficient was calculated as 0.31, indicating that for each unit increase in microvessel density (MVD), the odds ratio decreases by 31%. Additionally, the distribution of anastomotic leakage cases is illustrated in Fig. 1C, along with the logistic regression function. The optimal cutoff value was determined to be 0.9216 using Youden’s index. Subsequently, the study cohort was divided into two groups based on this cutoff value.

Multivariate frequency distributions were analyzed using Fisher’s exact test, and for numeric data, differences between the two groups were identified by an unpaired t-test with Welch’s correction. Regression analysis was performed using the generalized linear model. Univariable regression analyses were conducted to assess the effect of individual independent variables on the dependent variable. In cases where multiple independent variables appeared to influence the dependent variable, multivariable regression analyses were performed to evaluate their combined impact.

## Results

### Patient stratification

79 patients who underwent two-stage esophagectomy between 2016 and 2021 at our institution were included in the analysis. Figure 1A depicts the distribution of patients over different values of microvessel density (MVD) in the mucosa of the gastric ring, with an average MVD of 1.194% (± 0.059% SEM). Figure 1B shows representative images of CD34 stained sections. To test the hypothesis that gastric mucosal vascularity impacts postoperative outcome measures after esophagectomy, we stratified patients into two groups. Using the optimal cutoff value calculated by the Youden Index from a logistic regression model for the binary outcome parameter “anastomotic leakage” (Fig. 1C), patients were divided into a low MVD group (*n*=31) and a high MVD group (*n*=48).

Demographic profiles were comparable across the two groups (Table 1). Comorbidities were also similar between the two groups except of a difference in congestive heart failure (Supplementary Table 1). The indication for two-stage esophagectomy was established prior to the procedure, with 57 patients undergoing the two-step approach due to vascular (72.15%) and 22 patients due to pulmonary (27.85%) indications. Time between the first step of gastric mobilization and completion of esophagectomy was within a timespan from 1 to 7 days. Median time to reconstruction was 4 days in the high MVD group (± 1.00) and 4 days for the low MVD group (± 1.19) for each group (Table 1).

**Table 1:** General characteristics

Characteristics	Total		Low MVD		High MVD		*P*-value
	*n*=79	%	*n*=31	%	*n*=48	%	
Age (years, mean ±SD)	68.6 ± 9.03		68 ± 8.59		69.04 ± 9.54		0.616
*Sex*							0.999
Male	69	87.34	27	87.10	41	85.42	
Female	10	12.66	4	12.90	7	14.58	
*ASA*							0.659
1	4	5.06	1	3.23	3	6.25	
2	28	35.44	11	35.48	17	35.42	
3	47	59.49	19	61.29	28	58.33	
*ECOG*							0.609
0	25	31.65	12	38.71	13	27.08	
1	42	53.16	14	45.16	28	58.33	
2	10	12.66	4	12.90	6	12.50	
3	2	2.53	1	3.23	1	2.08	
*Histology*							0.777
AC	63	79.75	24	77.42	39	81.25	
SCC	16	20.25	7	22.58	9	18.75	
*Neoadjuvant therapy*							0.227
No	14	17.72	3	9.68	11	22.92	
Yes	66	83.54	28	90.32	37	77.08	
RCTX	40	61.54	17	60.71	23	62.16	0.999
CTX	25	38.46	11	39.29	14	37.84	
*Pathological T-stage*							0.854
pT0	14	17.72	7	22.58	7	14.58	
pT1	15	18.99	3	9.68	12	25.00	
pT2	8	10.13	3	9.68	5	10.42	
pT3	41	51.90	18	58.06	23	47.92	
pT4	1	1.27	0	0.00	1	2.08	
*Pathological N-stage*							0.584
pN0	38	48.10	15	48.39	23	47.92	
pN1	17	21.52	6	19.35	11	22.92	
pN2	12	15.19	3	9.68	9	18.75	
pN3	12	15.19	7	22.58	5	10.42	
*Resection margin*							0.384
R0	74	93.67	29	93.55	46	95.83	
R1	5	6.33	3	9.68	2	4.17	
*UICC-stadium*							0.482
I	22	27.85	8	25.81	14	29.17	
II	11	13.92	4	12.90	7	14.58	
III	35	44.30	13	41.94	22	45.83	
IV	11	13.92	6	19.35	5	10.42	
*Clinical T-stage*							0.074
uT1	5	6.33	0	0	5	10.42	
uT2	18	22.78	5	16.12	8	16.67	
uT3	56	70.89	26	83.87	35	72.92	
*Clinical N-stage*							0.999
uN0	5	6.33	2	6.45	3	6.25	
uN+/uNx	74	93.67	29	93.55	45	93.75	
*Time to reconstruction* (Median)	4 ±1.07		4 ± 1.19		4 ± 1.00		0.271
*Indication for 2 stage*							0.608
Vascular	57	72.15	21	67.74	36	75.00	
Pulmonal	22	27.85	10	32.26	12	25.00	

### High MVD correlated with a reduced risk of anastomotic leakage

Among 79 patients, the overall rate of anastomotic leakage was 12.66% (10 out of 79 patients). As we used the logistic regression model of anastomotic leakage and MVD to stratify into the two groups, accordingly, the high microvessel density (MVD) group exhibited a lower rate of anastomotic leakage compared to the low MVD group (6.25% *n*=3 vs. 22.58% *n*=7, *p*=0.043, Table 2).

**Table 2:** Frequency of anastomotic leakage

Anastomotic leakage	Total		Low MVD		High MVD		*P*-value
	*n*=79	%	*n*=31	%	*n*=48	%	
Yes	10	12.66	7	22.58	3	6.25	0.043
No	69	87.34	24	77.42	45	93.75	

To underscore the strength of MVD as potential predictor of anastomotic leakage we performed an univariable regression analysis with several covariates (smoking, diabetes, COPD, ASA score) that can be associated with anastomotic leakage. Patients with a high MVD had 77% lower odds for anastomotic leakage (OR 0.23, 95% CI 0.05–0.90, *p*= 0.045, Table 3)

**Table 3:** MVD as isolated risk factor of anastomotic leakage

Univariable regression anastomotic leakage	OR	*p*-value	95% confidence interval	
			Lower	Upper
ASA score	0.87	0.8	0.30	2.85
COPD	1.24	0.8	0.25	5.01
DM II	1.68	0.5	0.33	6.95
Smoking	1.61	0.5	0.44	6.50
MVD	0.23	0.045	0.05	0.90

Among patients undergoing two-stage esophagectomy for vascular indications, such as calcification of the thoracic aorta or stenosis of the celiac trunk, the anastomotic leakage rate was 33.33% in the low MVD group (7 out of 21 patients) compared to 8.33% in the high MVD group (3 out of 33) (*p*=0.028, Table 4). Notably, in the subgroup of patients that underwent a two-stage esophagectomy due to pulmonary indications no anastomotic leakage occurred.

**Table 4:** Frequency of anastomotic leakage in patients with a vascular indication

Anastomotic leakage by vascular indication	Total		Low MVD		High MVD		*P*-value
	*n*=57	%	*n*=21	%	*n*=36	%	
*Yes*	10	17.54	7	33.33	3	8.33	0.028
*No*	47	82.46	14	66.67	33	91.67	

### High MVD correlates with a reduced risk of postoperative complications

We hypothesized that, since anastomotic leakage largely contributes to major complications, MVD correlates with overall postoperative complications. Distribution of CD in dependence of the MVD is illustrated in Table 5. The CD score was lower in the high MVD group compared to low MVD (Median CD II vs. IIIa *p*=0.022, Table 5). Complications included pneumonia, delayed gastric conduit emptying (DGCE) with esophagogastroduodenoscopy and pneumatic pylorus dilatation, postoperative absolute tachyarrhythmia (TAA), pleural effusion with necessity of pleural drain, bleeding, wound healing disorder, respiratory insufficiency with reintubation, chylothorax, urinary tract infection, reoperation and recurrent laryngeal nerve injury (Supplementary Table 2).

**Table 5:** Distribution of Clavien Dindo scores

Clavien Dindo	Total		Low MVD		High MVD		*P*-value
	*n*	%	*n*	%	*n*	%	
0	15	18.99	4	12.90	11	22.92	0.022
I	7	8.86	1	3.23	6	12.50	
II	11	13.92	4	12.90	7	14.58	
IIIa	28	35.44	10	32.26	18	37.50	
IIIb	3	3.80	3	9.68	0	0.00	
IVa	5	6.33	3	9.68	2	4.17	
IVb	7	8.86	4	12.90	2	4.17	
V	4	5.06	2	6.45	2	4.17	

Patients with a high MVD showed a lower incidence of major complications (CD ≥ IIIb) in comparison to patients with a low MVD (12.5%, *n*=6 vs. 38.71%, *n*=12, *p*=0.031, Table 6). As multiple factors demonstrated an impact on major complications in the univariable regression analysis (Supplementary Table 3), a multivariable analysis was conducted. Here, high MVD was the only factor that showed reduced odds for major complications (OR 0.17, 95% CI 0.05-0.61, *p*= 0.008, Table 7) while smoking was the only factor with increased odds for major complications (OR 5.74, 95% CI 1.35-34, *p*= 0.03, Table 7). Among patients who developed anastomotic leakage (*n*=10), the majority were classified as Clavien-Dindo grade IIIa (*n*=6), indicating management via endoscopic intervention without any readmission to ICU (Table 8).

**Table 6:** High MVD correlate with lower rate of major complications

Major complications	Total		Low MVD		High MVD		*P*-value
	*n*=79	%	*n*=31	%	*n*=48	%	
Major complications (Clavien Dindo ≥IIIb)	18	22.78	12	38.71	6	12.50	0.031
Minor complications (Clavien Dindo ≤IIIa)	61	77.22	19	61.29	42	87.50

**Table 7:** MVD as risk factor of major complications

Multivariable regression major complications	OR	*p*-value	95% confidence interval	
			Lower	Upper
ASA score	1.21	0.8	0.36	4.48
COPD	3.1	0.093	0.82	12.0
DM II	0.57	0.5	0.10	2.56
Smoking	5.74	0.03	1.35	34
MVD	0.17	0.008	0.05	0.61

**Table 8:** Distribution of Anastomotic leakage and Clavien Dindo

Clavien Dindo	Anastomotic leakage	
	Yes	No
0	0	15
I	0	7
II	0	11
IIIa	6	22
IIIb	0	3
IVa	1	4
IVb	3	3
V	0	4

There was no difference between the two groups concerning hospital mortality (low MVD 6.45%, *n*=2, high MVD 4.17%, *n*=2, *p*=0.643, total *n*=4, 5.06%) (Supplementary Table 4).

### High MVD is associated with a shorter hospital stay

Additionally, a high MVD in the mucosa of the gastric ring showed a shorter overall hospital stay compared to low MVD patients (17.9 days (±12.57) vs. 23.1 days (±7.21), *p*=0.045, Table 9).

**Table 9:** Length of hospital stay

Length of hospital stay (lohs)	Total		Low MVD		High MVD		*P*-value
	*n*=79		*n*=31	%	*n*=48	SD	
Days ± SD	19.9	±9.92	23.1	±12.57	17.9	±7.21	0.045

## Discussion

The present work shows that poor vascularization of the stomach at the anastomotic site in patients undergoing two-stage esophagectomy for esophageal cancer correlates with an increased risk of anastomotic leakage, the occurrence of major complications, and prolonged hospital stay.

### Inter-patient variability in gastric vascularization

The first simple but relevant observation of our study is the fact that vascularization apparently differs from patient to patient. This is in line with data from the LOGIC Trial, which identified differences in baseline perfusion of the gastric fundus [20]. While various techniques have been used to assess gastric conduit microcirculation [21], this study is the first to histologically characterize vascularity in a large cohort. Histological staining the gastric ring provided a simple, inexpensive and reproducible method to measure conduit vascularity.

### Low MVD is correlated with AL

Statistical modeling stratified patients into a low and high MVD group leading to a low MVD group that showed a high rate of AL (22.58%) and a high MVD group that was associated with a very low rate of AL (6.25%). Though the current study does not provide any further evidence that a poor blood supply of the stomach at the anastomotic site directly results in AL we show a clear association between vascularization and clinical outcome that might be relevant in clinical practice. Previous work has demonstrated that gastric devascularization during esophagectomy significantly reduces perfusion at the anastomotic site [22] and that high-risk patients with a poor vascular status have a tremendously increased risk of anastomotic leakage [6].

As the present work explores the association between vascularity and outcome in high-risk patients of whom a significant amount had vascular co-morbidities it is reasonable to conclude or at least see our work as further evidence that the association of calcification of gastric arteries and anastomotic leakage as described by others is a result of a poor microcirculation.

### Low MVD is associated with major complications and a shorter hospital stay

Additionally, patients with a high MVD had a lower incidence (12.5%) of major complications (CD ≥ IIIb) in comparison to patients with a low MVD (38.71%). In the entire collective (*n*=79) 10 patients suffered from anastomotic leakage. 6 of those patients did not have major complications according to CD as they were treated endoscopically without any other intervention, surgery or ICU re-admission, which corresponds with CD IIIa. Accordingly, anastomotic leakage contributed with 4 patients to a total of 18 patients (22.8%). This could suggest that not only anastomotic leakage but other complications in the low MVD group contribute to the increased rate of major complications agreeing with the fact that patients in the low MVD group showed a trend towards more postoperative pneumonia and e.g., more reintubations though these differences were not statistically significant.

Not surprisingly fewer AL and fewer major complications translated into a shorter hospital stay in patients with a high MVD. This altogether suggests that a high MVD is associated with a less complicated and shorter hospital stay which presumably results in faster functional recovery and less health care costs. Whether this holds true beyond two-stage esophagectomy requires further investigation.

### Neoadjuvant therapy and MVD

In our cohort, although these changes were not significant, patients with a high MVD underwent less often neoadjuvant treatment. The link between neoadjuvant therapy and AL remains debated, with a recent meta-analysis finding no correlation, especially with radiation [23]. However, it is possible that patients without neoadjuvant therapy experienced fewer complications or that such treatment reduced gastric MVD, contributing to AL.

## Conclusion

This study uncovers an association between vascularity of the stomach at the anastomotic site and reduced postoperative morbidity after two-stage Ivor-Lewis esophagectomy. MVD may serve as a novel biomarker for identifying high-risk patients, warranting validation through prospective trials and further translational research.

## Supplementary Information

Below is the link to the electronic supplementary material.

Supplementary file1 (DOCX 22 KB)
