# Supplementary material for: Vascularity of the gastric conduit predicts complications after Ivor-Lewis esophagectomy
Source: Surg Endosc. 2025 May 8;39(6):3839–47. doi: 10.1007/s00464-025-11780-8 (PMC12116690; doi:10.1007/s00464-025-11780-8)
Supplement: Supplementary file 1 — Supplementary file1 (DOCX 22 KB) [file 464_2025_11780_MOESM1_ESM.docx]

## Supplementary Table 1

| Comorbidities | TOTAL | | Low MVD | | High MVD | | P-Value |
| --- | --- | --- | --- | --- | --- | --- | --- |
|  | n | % | n | % | n | % |  |
| Arterial hypertension |  |  |  |  |  |  | 0.628 |
| Yes | 52 | 65.82 | 19 | 61.29 | 33 | 68.75 |  |
| No | 27 | 34.18 | 12 | 38.71 | 15 | 31.25 |  |
| Congestive heart failure |  |  |  |  |  |  | 0.021 |
| Yes | 16 | 20.25 | 3 | 9.68 | 13 | 27.08 |  |
| No | 54 | 68.35 | 29 | 93.55 | 25 | 52.08 |  |
| Atrial fibrillation |  |  |  |  |  |  | 0.597 |
| Yes | 18 | 22.78 | 6 | 19.35 | 12 | 25.00 |  |
| No | 61 | 77.22 | 25 | 80.65 | 36 | 75.00 |  |
| Coronary artery disease |  |  |  |  |  |  | 0.732 |
| Yes | 10 | 12.66 | 3 | 9.68 | 7 | 14.58 |  |
| No | 69 | 87.34 | 28 | 90.32 | 41 | 85.42 |  |
| History of myocardial infarction |  |  |  |  |  |  | 0.395 |
| Yes | 6 | 7.59 | 1 | 3.23 | 5 | 10.42 |  |
| No | 73 | 92.41 | 30 | 96.77 | 43 | 89.58 |  |
| COPD |  |  |  |  |  |  | 0.116 |
| Yes | 20 | 25.32 | 11 | 35.48 | 9 | 18.75 |  |
| No | 59 | 74.68 | 20 | 64.52 | 39 | 81.25 |  |
| FEV1 > 80% |  |  |  |  |  |  | 0.354 |
| Yes | 34 | 43.04 | 11 | 35.48 | 23 | 47.92 |  |
| No | 45 | 56.96 | 20 | 64.52 | 25 | 52.08 |  |
| Total FEV1  (l/min, mean ± SD) | 2.62 ± 0.63 | | 2.61 ± 0.63 | | 2.63 ± 0.62 | | 0.993 |
| VC > 80% |  |  |  |  |  |  | 0.810 |
| Yes | 28 | 35.44 | 10 | 32.26 | 18 | 37.50 |  |
| No | 51 | 64.56 | 21 | 67.74 | 30 | 62.50 |  |
| Total VC (l, mean ± SD) | 3.5 ± 0.65 | | 3.44 ± 0.68 | | 3.55 ± 0.64 | | 0.555 |
| Peripheral artery diseases |  |  |  |  |  |  | 0.793 |
| Yes | 12 | 15.19 | 7 | 22.58 | 5 | 10.42 |  |
| No | 67 | 84.81 | 24 | 77.42 | 43 | 89.58 |  |
| Diabetes |  |  |  |  |  |  | 0.999 |
| Yes | 21 | 26.58 | 8 | 25.81 | 13 | 27.08 |  |
| No | 58 | 73.42 | 23 | 74.19 | 35 | 72.92 |  |
| Smoking |  |  |  |  |  |  | 0.802 |
| Yes | 57 | 72.15 | 23 | 74.19 | 34 | 70.83 |  |
| No | 22 | 27.85 | 8 | 25.81 | 14 | 29.17 |  |
| Pack years (mean ± SD) | 42.31 ± 24.88 | | 45.3 ± 25.42 | | 40 ± 24.16 | | 0.462 |

## Supplementary Table 2

| Complications | TOTAL | | Low MVD | | High MVD | | P-Value |
| --- | --- | --- | --- | --- | --- | --- | --- |
|  | n=79 | % | n=31 | % | n=48 | % |  |
| Pneumonia |  |  |  |  |  |  | 0.305 |
| Yes | 22 | 27.85 | 11 | 35.48 | 11 | 22.92 |  |
| No | 57 | 72.15 | 20 | 64.52 | 37 | 77.08 |  |
| DGCE |  |  |  |  |  |  | 0.777 |
| Yes | 16 | 20.25 | 7 | 22.58 | 9 | 18.75 |  |
| No | 63 | 79.75 | 24 | 77.42 | 39 | 81.25 |  |
| Post OP TAA |  |  |  |  |  |  | 0.784 |
| Yes | 18 | 22.78 | 8 | 25.81 | 10 | 20.83 |  |
| No | 61 | 77.22 | 23 | 74.19 | 38 | 79.17 |  |
| Chest drain for pleural effusion |  |  |  |  |  |  | 0.489 |
| Yes | 36 | 45.57 | 16 | 51.61 | 20 | 41.67 |  |
| No | 43 | 54.43 | 15 | 48.39 | 28 | 58.33 |  |
| Bleeding |  |  |  |  |  |  | 0.392 |
| Yes | 1 | 1.27 | 1 | 3.23 | 0 | 0.00 |  |
| No | 78 | 98.73 | 30 | 96.77 | 48 | 100.00 |  |
| Wound healing disorder |  |  |  |  |  |  | 0.999 |
| Yes | 2 | 2.53 | 1 | 3.23 | 1 | 2.08 |  |
| No | 77 | 97.47 | 30 | 96.77 | 47 | 97.92 |  |
| Reintubation |  |  |  |  |  |  | 0.155 |
| Yes | 16 | 20.25 | 9 | 29.03 | 7 | 14.58 |  |
| No | 63 | 79.75 | 22 | 70.97 | 41 | 85.42 |  |
| Chylothorax |  |  |  |  |  |  | 0.057 |
| Yes | 3 | 3.79 | 3 | 9.68 | 0 | 0.00 |  |
| No | 76 | 96.20 | 28 | 90.32 | 48 | 100.00 |  |
| Urinary tract infection |  |  |  |  |  |  | 0.204 |
| Yes | 6 | 7.59 | 4 | 12.90 | 2 | 4.17 |  |
| No | 75 | 94.93 | 27 | 87.10 | 46 | 95.83 |  |
| Reoperation for any reason |  |  |  |  |  |  | 0.367 |
| Yes | 5 | 6.32 | 4 | 12.90 | 2 | 4.17 |  |
| No | 73 | 92.40 | 27 | 87.10 | 48 | 95.83 |  |
| Recurrent laryngeal nerve injury |  |  |  |  |  |  | 0.999 |
| Yes | 1 | 1.26 | 0 | 0.00 | 1 | 2.08 |  |
| No | 78 | 98.73 | 31 | 100.00 | 47 | 97.92 |  |

## Supplementary Table 3

| Univariable regression major complications | OR | p-value | 95 % confidence interval | |
| --- | --- | --- | --- | --- |
|  |  |  | lower | upper |
|  |  |  |  |  |
| ASA score | 1.04 | >0.9 | 0.44 | 2.70 |
| COPD | 4.27 | 0.012 | 1.38 | 13.60 |
| DM II | 0.67 | 0.60 | 0.14 | 2.41 |
| Smoking | 4.52 | 0.022 | 1.40 | 20.3 |
| **MVD** | **0.17** | **0.008** | **0.05** | **0.61** |

## Supplementary Table 4

| Hospital mortality | TOTAL | | Low MVD | | High MVD | | P-Value |
| --- | --- | --- | --- | --- | --- | --- | --- |
|  | n=79 | % | n=31 | % | n=48 | % |  |
|  | 4 | 5.06 | 2 | 6.45 | 2 | 4.17 | 0.643 |
